# Light driven water oxidation on silica supported NiO–TiO_2_ heteronanocrystals yields hydrogen peroxide†

**DOI:** 10.1039/d4na00906a

**Published:** 2025-02-10

**Authors:** Nurul Muttakin, Shelton J. P. Varapragasam, Rashed Mia, Mahfuz A. Swadhen, Michael Odlyzko, James D. Hoefelmeyer

**Affiliations:** a Department of Chemistry, University of South Dakota Vermillion SD 57069 USA james.hoefelmeyer@usd.edu; b Characterization Facility, University of Minnesota Minneapolis MN 55455 USA

## Abstract

Decomposition of nickel nitrate hexahydrate in the presence of rod-shape anatase TiO_2_ nanocrystals led to the formation of NiO–TiO_2_ heteronanocrystals confirmed with powder X-ray diffraction and electron microscopy. The heteronanocrystals were supported on amorphous fumed silica to provide a heterogeneous photocatalyst material SiO_2_/NiO–TiO_2_. The aqueous suspension of the catalyst, under argon atmosphere, irradiated with a Xe arc lamp led to the formation of H_2_O_2_ with trace gaseous product formation. We observed an initial rate of formation of H_2_O_2_ of 1.8 μmol g^−1^ min^−1^ that decays toward a steady-state concentration of 52 μM. Addition of AgNO_3_ to the aqueous suspension gave fast reduction of silver ion, and higher initial rates of formation and steady state concentrations of H_2_O_2_. We report the concentration dependence of water oxidation *versus* [AgNO_3_] with the fastest initial rate of formation of H_2_O_2_ as 9.1 μmol g^−1^ min^−1^ and a steady-state concentration of 174 μM.

## Abstract 2

Light illumination of aqueous suspensions of supported NiO–TiO_2_ heteronanocrystals led to water oxidation to form hydrogen peroxide.

## Graphical abstract

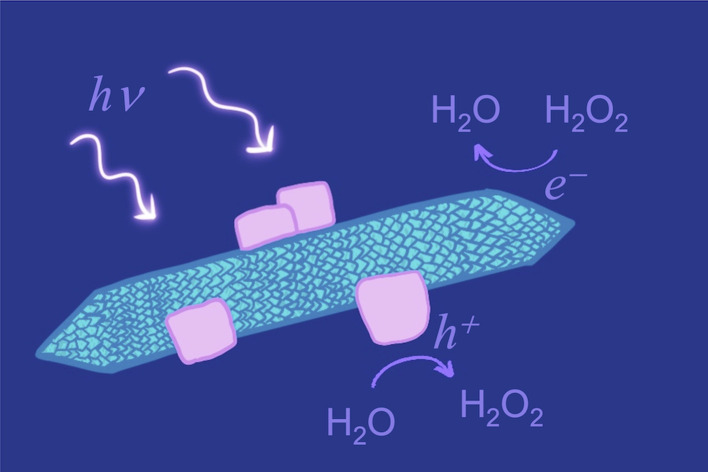

## Introduction

There is longstanding interest in new materials for light driven catalytic processes, especially those that may use sunlight.^1–10^ In addition to applications such as water purification,^11–13^ pollution remediation,^14,15^ or self-cleaning windows,^16^ the prospect of capturing and converting light energy to make fuels (solar fuels) could provide an important option for the energy intensive global economy.^17,18^ Much inspiration for the design of photocatalyst materials originates in the study of photosynthesis.^19–24^ Natural photosynthesis occurs in a highly preorganized multistep process that is difficult to achieve in artificial systems. However, key steps occur in both cases: electronic excitation of a chromophore upon absorption of a photon of energy, fast electron transfer steps that disrupt rapid relaxation of the chromophore to the ground state, and fast redox catalytic steps to convert the harvested reducing/oxidizing equivalents to stable fuel/oxidizer molecules.

**Scheme 1:**
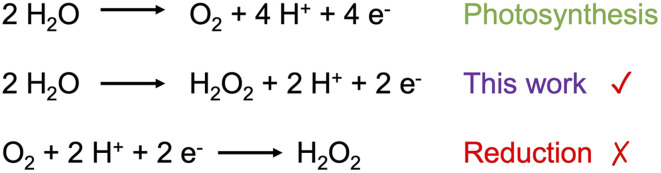
Water oxidation to hydrogen peroxide in comparison to water oxidation to molecular oxygen or oxygen reduction to hydrogen peroxide.

In natural photosynthesis, the oxidation pathway (Scheme 1) is four-electron water oxidation that is endothermic and kinetically challenging.^25,26^ As an evolutionary step, this pathway has the advantages of utilizing an abundant feedstock, water, and does not produce toxic biproducts. For instance, one-electron oxidation of hydroxide anion or a net two-electron water oxidation step to hydrogen peroxide would yield reactive products toxic to the cell. Artificial photosynthesis systems do not have this restriction, and there may be significant advantages in alternative water oxidation approaches, such as faster reaction kinetics and more valuable oxidation products.^27,28^ For example, hydrogen peroxide can be used for metal-peroxide batteries,^29^ disinfectant, or bleaching agent.^30–33^ Though hydrogen peroxide can be formed by oxygen reduction pathways, including photocatalytic systems, the utility of hydrogen peroxide from water oxidation could be a significant industrial advantage when coupled with productive reduction pathways.^34^ Recently, catalyst materials for light-driven water splitting yielding hydrogen and hydrogen peroxide have been reported.^35–39^

The economic viability of solar fuels catalysis improves with low-cost materials and processing, non-toxic and robust materials, and high catalytic activity. Many of the materials developed for solar fuels catalysis use semiconductors modified to improve light absorption, electron/hole separation, and catalysis.^40,41^ Metal oxides can satisfy many of these criteria.^10,42^ We focus on using nanostructured TiO_2_ because it has important advantages of low cost, ease and variety of synthetic methods that afford a wide range of nanoscale morphology, low toxicity, high stability, and electronic structure that permits important redox processes in aqueous solution. The important limitations of TiO_2_ in solar fuels catalysis applications are that its large bandgap limits light absorption to the UV spectrum, internal conversion occurs on a timescale of picoseconds, and electron transfer catalysis on the TiO_2_ surface is slower than internal conversion by several orders of magnitude.^43^ Much research over the last few decades addresses these problems.^44^ Here we focus on the cocatalyst attached to the semiconductor through preparation of heteronanocrystals.

We prepared NiO–TiO_2_ heteronanocrystals consisting of rod-shape anatase TiO_2_ multiply decorated with NiO. We adopt the rod-shape morphology of the semiconductor TiO_2_ due to the longitudinal electron transfer with little energy loss that has been invoked as a strategy to assist in separating photo-generated electron/hole pairs.^45^ The NiO–TiO_2_ interface is a type II p/n heterojunction in which TiO_2_ is n-type and NiO is p-type.^46^ This electronic configuration promotes separation of e^−^/h^+^ pairs wherein the electron becomes localized on TiO_2_ and the hole becomes localized on NiO.^47–58^ Furthermore, thin films of NiO, Ni(OH)_2_, NiOOH materials have been shown as effective electrocatalysts for water oxidation reactions that tend to be a kinetic bottleneck in solar fuels catalysis.^59–61^ We report the preparation and characterization of the NiO–TiO_2_ heteronanocrystal and evaluate aqueous photocatalysis of the silica supported heteronanocrystal SiO_2_/NiO–TiO_2_. Importantly, this material exhibits fast water oxidation with formation of H_2_O_2_ in aqueous solution.

## Experimental

### Chemicals and equipment

Titanium(iv)tetraisopropoxide (98+%, Acros Organics), oleic acid (90%, Fisher Scientific), 1-octadecene (technical grade, 90%, Acros), oleylamine (>50%, TCI America), trioctylphosphine, (technical grade, 90% AcroSeal™, Thermo Scientific™), Ni(NO_3_)_2_·6H_2_O (J.T. Baker chemical Co.), AgNO_3_ (99%, VWR), 1,4-benzoquinone (TCI America), ferrous sulfate heptahydrate (Fisher scientific), methanol (99.9%, HPLC grade, 0.2 μm filtered, Fisher Scientific), Pierce™ Quantitative Peroxide Assay Kits (ThermoFisher), potassium iodide (99+%, Acros Organics), ammonium dimolybdate (Beantown Chemical), hydrogen peroxide (30%, Acros organics), nylon syringe filter with 0.45 μm pore size (Tisch), and 96 well microplate (Greiner) were used. TiO_2_ nanorods and NiO–TiO_2_ HNCs were synthesized under a flow of N_2_ with an in-line drying tower charged with P_2_O_5_. Sonication procedures utilized a Neytech 28H Ultrasonik (100 W, 44–48 kHz) sonication bath. Vortex mixing was achieved with a Vornado benchtop vortex mixer. Calcinations utilized an MTI KSL-1100X furnace. Centrifugation protocols utilized benchtop Thermo IEC Centra CL-2 or Eppendorf 5804 models configured to accept Falcon 50 mL conical centrifuge tubes. Labnet Prism Air-Cooled Microcentrifuge C2500 with 24-Place Microtube Rotor were also utilized to accept BIOLOGIX 1.5 mL microcentrifuge tubes.

### Instrumentation

Transmission electron microscopy (TEM) images were obtained from a Tecnai Spirit G^2^ Twin TEM (FEI Company) with a LaB_6_ filament operating at 120 kV. HR-TEM images were obtained from a ThermoFisher Talos F200X G^2^ with X-FEG Schottky FEG (ThermoFisher) electron source. STEM detector model (Fischione Model 3000 HAADF, ThermoFisher DF4, ThermoFisher DF2, ThermoFisher BF), X-ray detector model with solid angle 0.9 sr (ThermoFisher Super-X G2) and electron source brightness (1.8 × 10^9^ A cm^−2^ sr^−1^) were used. Experiments were performed with a beam current of 11 nA for CTEM and 0.3 nA for STEM. Powder X-ray diffraction (PXRD) data were obtained from a Rigaku Ultima IV instrument. The X-ray tube produced Cu Kα radiation (*λ* = 1.54 Å), and the generator was set to 44 kV and 44 mA during data collection. Data refinement and analysis were performed with PDXL software. A Cary 50 spectrophotometer was used to obtain UV-visible absorbance spectra of nanocrystal dispersions in hexanes. Photoluminescence spectra were obtained using a Fluoromax-4 spectrofluorometer. Hydrogen generation in the headspace of the photochemical reactor was measured by gas chromatography (GC) using an Agilent 7890A GC equipped with a 30 m × 320 μm column with 5 Å molecular sieves and Ar as the carrier gas.

### Synthesis of anatase TiO_2_ nanorods

Anatase TiO_2_ nanorods with different aspect ratios were synthesized using non-hydrolytic sol–gel ester elimination reaction under inert condition according to the synthetic procedure developed by the Hyeon group.^62^ Degassed 52.5 g oleic acid and 16.95 g (=17.65 mL) titanium isopropoxide (TTIP) (60 mmol) were heated under inert conditions (with venting of *in situ* formed isopropanol) until reaching a temperature of 270 °C and was kept for 2 hours. The reaction was allowed to cool down to room temperature and 120 mL isopropanol was added to isolate the product. The reaction mixture was centrifuged at 3500 rpm for 8 minutes and the supernatant was discarded. Size selective precipitation: the product was redispersed in 60 mL hexane to which 25 mL of isopropanol was added and centrifuged at 11 000 rpm for 3 minutes to collect the first fraction of TiO_2_ nanorods. The first fraction of TiO_2_ nanorods were redispersed in 20 mL hexane. To remove any residual nanospheres, 20 mL isopropanol was added and the contents were centrifuged at 11 000 rpm for 3 minutes. Finally, 20 mL of hexane was used to redisperse exclusively TiO_2_ nanorods.

### Synthesis of binary phase NiO–TiO_2_ heteronanocrystals

In this synthesis, 0.068 g (0.85 mmol) (inorganic mass) of pre-synthesized TiO_2_ nanorods dispersed in hexane was transferred into a 100 mL three-neck flask with 11.2 mL oleylamine, 20 mL 1-octadecene, 9.2 mL trioctylphosphine, and 0.996 g (3.4 mmol) of Ni(NO_3_)_2_·6H_2_O. The hexane in the reactant mixture was removed gradually under vacuum at room temperature and then heated to 50 °C for 20 minutes under vacuum. After removal of the volatiles, the flask was filled with nitrogen. The contents were heated to 230 °C at 10 °C min^−1^ and kept at that temperature for 5 minutes. The reaction was stopped by removing the heating mantle. Once the reaction mixture cooled down to room temperature, 80 mL isopropanol was added. The contents were centrifuged at 3500 rpm for 8 minutes, and the supernatant was discarded. The precipitate was dispersed in 10 mL hexane.

### Preparation of supported catalysts: SiO_2_/TiO_2_ and SiO_2_/NiO–TiO_2_

A slurry of 0.667 g fumed silica with 40 mL of hexanes was prepared in a 50 mL centrifuge tube. The contents were agitated with a vortex mixer for 2 min. Then, 20 mg of photocatalyst (TiO_2_ or NiO–TiO_2_) dispersed in hexanes was added to the centrifuge tube and sonicated for 20 min. After that, the contents were centrifuged at 3500 rpm for 8 min, and the clear supernatant was discarded. The uptake of TiO_2_ on the SiO_2_ surface was verified by UV-visible spectroscopy (supernatant was absent the distinct O → Ti charge transfer feature). The gelatinous precipitate of SiO_2_/NiO–TiO_2_ was spread onto a watch glass and dried under vacuum (∼30 mtorr) at 175 °C for 24 h. The sample was allowed to cool to room temperature and was ground with a mortar and pestle.

### Photocatalysis studies

A 150 mL quartz flask with a magnetic stirrer was charged with 10 mL of water and 167 mg catalyst. The flask was sealed with a rubber septum and fitted with a 2.5′′ 20 gauge needle outlet. Argon gas (∼50 sccm) was introduced to the flask through a needle to purge the contents for 30 min, then the inlet and outlet needles were removed. Using a gastight syringe, a 1 mL aliquot of the headspace was extracted and injected into the GC, at which time no oxygen was detected. The flask was irradiated with a 150 W Xe Arc lamp powered by an ABET Technologies solar simulator. The photocatalytic reactor was covered with a wooden shield box to isolate the system from the outside light as well as to avoid UV-light exposure. The headspace was sampled at desired time intervals for GC analysis.

### Detection and quantification of photocatalytically generated H_2_O_2_

#### Detection with pierce quantitative peroxide assay kits

Detection of H_2_O_2_ generated form photocatalysis was determined by Pierce™ quantitative peroxide assay kits. A 1 mL aliquot was collected from the reaction vessel and was subjected to centrifugation at 8000 rpm at 5 min to remove Ag nanoparticles and catalysts. To further remove any residual catalysts and Ag nanoparticles, it was passed through a syringe filter with a pore size of 0.45 μm. Working reagent was prepared by mixing 1 volume of Reagent A with 100 volumes of Reagent B. 20 μL of purified aliquot was mixed with 200 μL of premixed working reagent (WR) in a in a 96-well plate and incubated for 4 h to complete the reaction. Also, 20 μL of deionized water is added in 200 μL of premixed WR in 96 well plate to run control experiment. The plate was placed in plate reader to collect the absorbance at 585 nm.

#### Quantification of photocatalytically generated H_2_O_2_

The concentration of H_2_O_2_ was determined using the Ghormley triiodide method.^63^ In this process, H_2_O_2_ causes the oxidation of I^−^ to I_3_^−^. The product's absorbance is then spectrophotometrically measured at 360 nm. A 600 μL aliquot was extracted from the reaction vessel at desired interval with the aid of a syringe. The extracted aliquot was subjected to centrifugation at 8000 rpm for 5 min to remove Ag nanoparticles and catalysts. To further remove any residual catalysts and Ag nanoparticles, it was passed through a syringe filter with a pore size of 0.45 μm. A stock solution was made to detect H_2_O_2_ with 800 μL deionized water, 50 μL 1 M acetic acid/sodium acetate, 50 μL 1 M potassium iodide, and 20 μL Ammonium dimolybdate. 100 μL purified aliquot was added to the stock solution to initiate the reaction. After allowing the solution to react for 5 minutes, the solution was centrifuged for 5 min at 8000 rpm and passed through a 0.45 μm syringe filter to remove AgI. Finally, 200 μL of aliquot was placed in 96 well plate to collect absorbance at 360 nm.

## Results and discussion

Rod shape anatase TiO_2_ nanocrystals were prepared according to the procedure developed by Hyeon.^62^ Powder X-ray diffraction (PXRD) data indicate anatase TiO_2_ with 97% crystallinity and no other phases present. The (004) reflection shows high relative intensity and narrow lineshape that indicate a highly anisotropic crystal consistent with elongation along the *c*-axis. Transmission electron microscopy (TEM) data confirm the highly anisotropic rod shape of the nanocrystal (length = 44.8 ± 8.2 nm, diameter = 3.0 ± 0.4 nm) and confirms the crystal growth along the *c*-axis, consistent with the prior literature.^62,64^ Williamson–Hall size analysis of powder X-ray diffraction data indicates a crystal size of 3.4 nm, which is in close agreement with the diameter found from TEM. The sample is well replicated in multigram quantities, and size selective precipitation techniques are required to obtain samples with uniform aspect ratio.

**Fig. 1:**
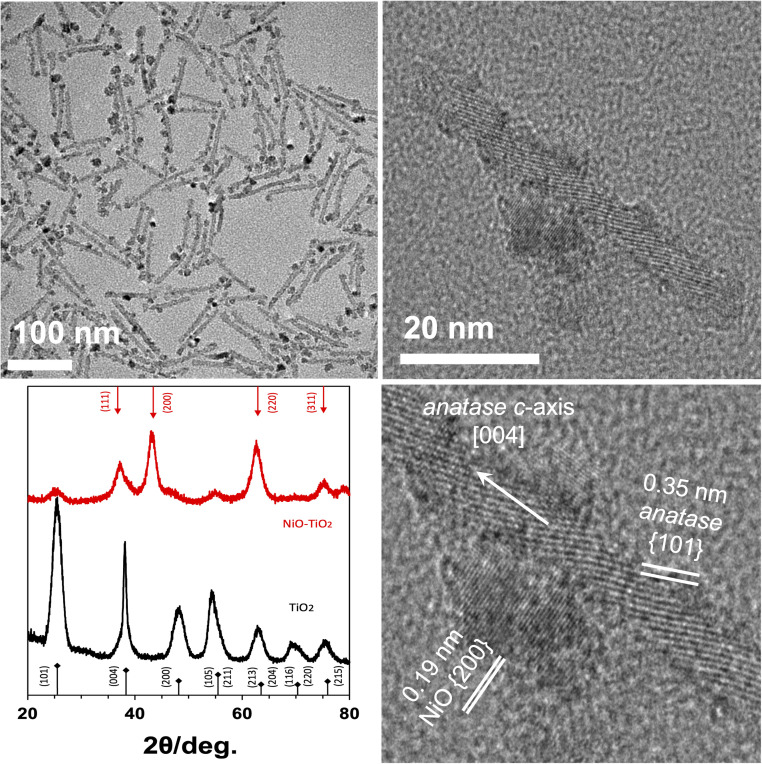
Clockwise from upper left: TEM image of NiO–TiO_2_ heteronanocrystals; HRTEM image of single heteronanocrystal; annotated closeup HRTEM; and PXRD patterns of TiO_2_ nanorods (black) and NiO–TiO_2_ heteronanocrystal (red) samples. Reference relative intensities for anatase^68^ TiO_2_ and bunsenite^69^ NiO are shown along bottom and top.

Decomposition of Ni(NO_3_)_2_·6H_2_O was carried out in 1-octadecene containing the TiO_2_ nanorods, oleylamine, and trioctylphosphine. The product was characterized (Fig. 1) with electron microscopy, X-ray diffraction, and spectroscopic methods (UV-visible absorbance and photoluminescence spectra: ESI, Fig. S1†). Powder X-ray diffraction data of samples obtained from the reaction indicated the presence of cubic bunsenite NiO and anatase TiO_2_ phases. Data analysis using the Rietveld and reference intensity ratio (RIR) indicated NiO and anatase TiO_2_ phases in a 77 : 23% ratio. However, given the differences in mass attenuation between NiO and TiO_2_, the analysis overestimates the proportion of NiO in the sample. Mass attenuation coefficients for O, Ti, and Ni may be found in NIST Standard Reference Database 126.^65^ The attenuated X-ray intensity is inversely proportional to the mass attenuation coefficient of the solid.^66^ Using values of 11.6, 202, and 49.5 cm^2^ g^−1^ at 8 keV (Cu K_α_) for O, Ti, and Ni, respectively, we calculate mass attenuation coefficients for NiO and TiO_2_ as 41.4 and 126 cm^2^ g^−1^. According to these, we estimate that the NiO phase is overestimated by a factor of 3 from uncorrected Rietveld analysis. Compensating for mass attenuation effects, the NiO and TiO_2_ phases are estimated as nearly equal abundance in the heteronanocrystal samples. Using the Williamson–Hall method, the sizes of the NiO and TiO_2_ crystallites were determined to be 3.2 and 2.9 nm, respectively. TEM micrographs show multiple domains of NiO attached to the TiO_2_ nanorods. The average diameter of NiO domains were 4.1 ± 0.8 nm. Though the synthesis conditions were reducing, we propose that air oxidation led to the observed NiO phase.^67^

**Fig. 2:**
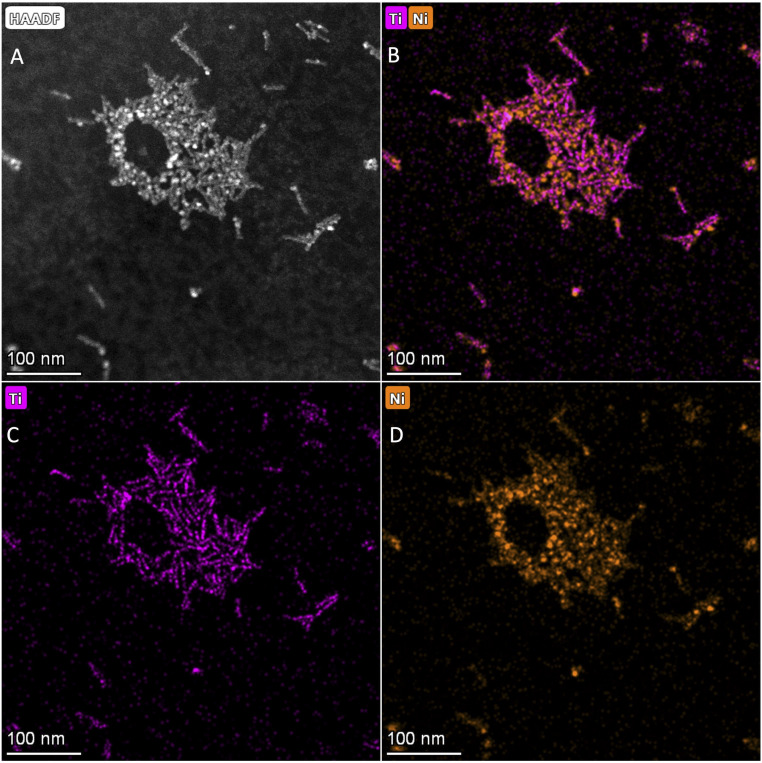
(A) HAADF-STEM image and (B) STEM-EDS mapping of TiO_2_-NiO HNCs, (C) STEM-EDS mapping of Ti, and (D) STEM-EDS mapping of Ni (scale bar = 100 nm).

Data obtained from HAADF and EDS analyses (Fig. 2) indicate heteronanocrystal structures. The HAADF images show bright regions dispersed among the less bright nanorods, consistent with NiO domains attached to TiO_2_ nanorods, while EDS data indicate distinct Ni rich domains on the Ti rich rod shape nanostructures. Quantitative EDS analysis (see ESI†) show ∼76 : 24% NiO : TiO_2_, consistent with powder X-ray diffraction analyses. The HRTEM data indicate the presence of crystalline cubic NiO attached to the anatase TiO_2_ nanorods. Selected HRTEM images of the NiO–TiO_2_ heteronanocrystal viewed through the [100] axis of the anatase nanocrystal show that the {200} plane of NiO is evident as well as the (101) and (004) planes of anatase TiO_2_. The NiO {200} plane is tilted by 3° from the (004) planes of anatase. Given the lattice parameter of 4.19 Å for NiO and *c* = 9.49 Å for anatase TiO_2_, the mismatch between NiO (200) and TiO_2_ (004) is 11.7%.

The heteronanocrystal surface is passivated with organic ligands, such as oleic acid, oleylamine, or trioctylphosphine. The hydrophobic ligands allow the heteronanocrystal to disperse rapidly in non-polar organic solvents. To study aqueous photocatalysis, we remove the organic ligands to liberate the surface sites on the heteronanocrystal. Our strategy was to immobilize the NiO–TiO_2_ on fumed silica, an inert support material, followed by heating in a (30 mtorr) vacuum oven at 175 °C for 24 hours to yield the photocatalyst material, SiO_2_/NiO–TiO_2_.

In distilled water, a SiO_2_/NiO–TiO_2_ suspension was exposed to unfiltered light from a Xe arc lamp. The headspace of the reactor was sampled with a 1 mL gas-tight syringe and analyzed by gas chromatography (GC). Under these conditions, we detected trace quantities of adventitious air and only trace amounts of hydrogen. We then attempted the irradiation of the catalyst in 10% methanol : water solution and found the same result from GC analysis. The results contrast with the known hydrogen evolution performance of TiO_2_ photocatalysts in water or methanol : water solutions.^70^ We then attempted a photoreaction with SiO_2_/NiO–TiO_2_ in water with silver nitrate, a well-established electron scavenger. In this case we observed rapid darkening of the solution due to formation of metallic silver (ESI: Fig. S2†). Control experiments in which aqueous silver nitrate was irradiated without catalyst did not lead to the formation of metallic silver. Suspensions of the catalyst in aqueous silver nitrate kept in the dark do not lead to silver reduction. Furthermore, fumed silica is inactive toward reduction of silver nitrate in the light or dark. The observations establish that the irradiated catalyst gives rise to the reduction of Ag^+^ that we hypothesize as due to photogenerated excitons, followed by electron localization on TiO_2_ concomitant with hole localization on NiO, and finally electron transfer from TiO_2_ to silver. Under these conditions hydrogen was absent, in contrast to prior experiments, which is consistent with Ag^+^ reduction being favored over H^+^ reduction. Certainly, reduction cannot occur without complimentary oxidation to give an electron balance in the system. This led us to consider other oxidation products. A commercially available Pierce™ quantitative hydrogen peroxide assay kit was used to analyze the aqueous solution with the result that hydrogen peroxide was formed in the photochemical reaction and its time-dependent concentration could be quantified. Similarly, a test based on the conversion of iodide to triiodide in the presence of ammonium dimolybdate also gave positive results consistent with formation of hydrogen peroxide in the aqueous solution after irradiation. These results immediately suggested to us that water oxidation to hydrogen peroxide on the NiO surface was fast under light irradiation.

**Fig. 3:**
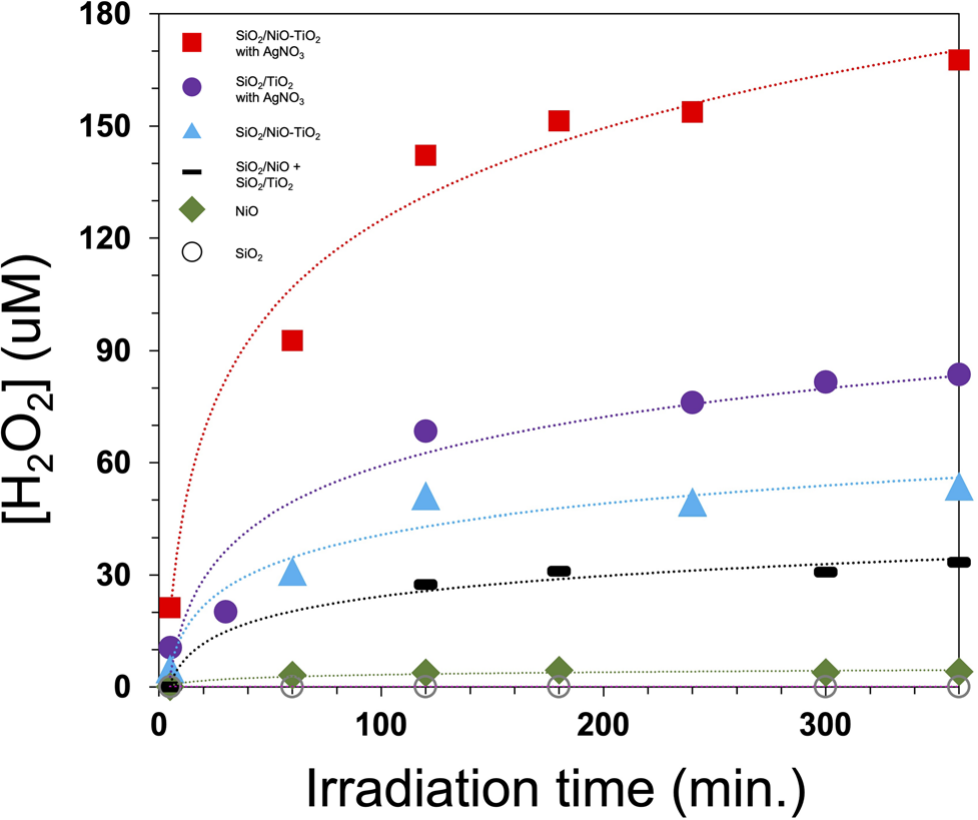
Plot of [H_2_O_2_] *vs.* time under irradiation of aqueous suspension of catalyst. Legend: square = SiO_2_/NiO–TiO_2_ w/AgNO_3_; circle = SiO_2_/TiO_2_ w/AgNO_3_; triangle = SiO_2_/NiO–TiO_2_; dash = SiO_2_/NiO + SiO_2_/TiO_2_; diamond = NiO; open circle = SiO_2_.

Fig. 3 shows the concentration of H_2_O_2_*versus* time found by *ex situ* testing of aliquots taken during the irradiation of aqueous suspensions containing catalyst materials with or without silver nitrate. Fumed silica was inactive and NiO alone showed rather low activity. A mixture of TiO_2_/SiO_2_ and NiO/TiO_2_ showed appreciable activity that can be attributed to the TiO_2_; TiO_2_/SiO_2_ alone showed similar activity. The formation of H_2_O_2_ from water oxidation on TiO_2_ has been reported,^63^ and theoretical studies indicate this pathway is favorable in comparison to four-electron water oxidation.^71,72^ We observe that the activity for the supported SiO_2_/NiO–TiO_2_ heteronanocrystal catalyst is substantially higher than SiO_2_/TiO_2_. Addition of AgNO_3_ to the reaction in which TiO_2_/SiO_2_ or SiO_2_/NiO–TiO_2_ was the catalyst led to higher activity in both cases. Generally, we observe a rapid increase in the concentration of H_2_O_2_ that gradually approaches a steady state concentration over time. This profile is consistent with forward and reverse reactions occurring simultaneously, one in which water oxidation leads to hydrogen peroxide and the reverse in which hydrogen peroxide undergoes reductive decomposition to water. Diesen and Jonsson noted this type of behavior on TiO_2_ nanoparticle films.^63^ In the case of the NiO–TiO_2_ heteronanocrystal, both the initial rate of hydrogen peroxide formation and its steady state concentration are greater than found for TiO_2_. The enhancement in activity is consistent with exciton separation at the type-II p/n heterojunction followed by fast oxidation of water to hydrogen peroxide on the NiO surface. In the absence of a fast electron scavenger, we propose that the freshly formed hydrogen peroxide is immediately reduced on the TiO_2_ surface. This leads to a closed chemical cycle with no net products and is consistent with several observations: initial rapid formation of H_2_O_2_, observation of steady-state concentration of H_2_O_2_, no oxygen detected, and zero-to-trace hydrogen detected (again, we note our observation of hydrogen in the absence of silver nitrate; when silver nitrate is present, we do not detect hydrogen).

**Fig. 4:**
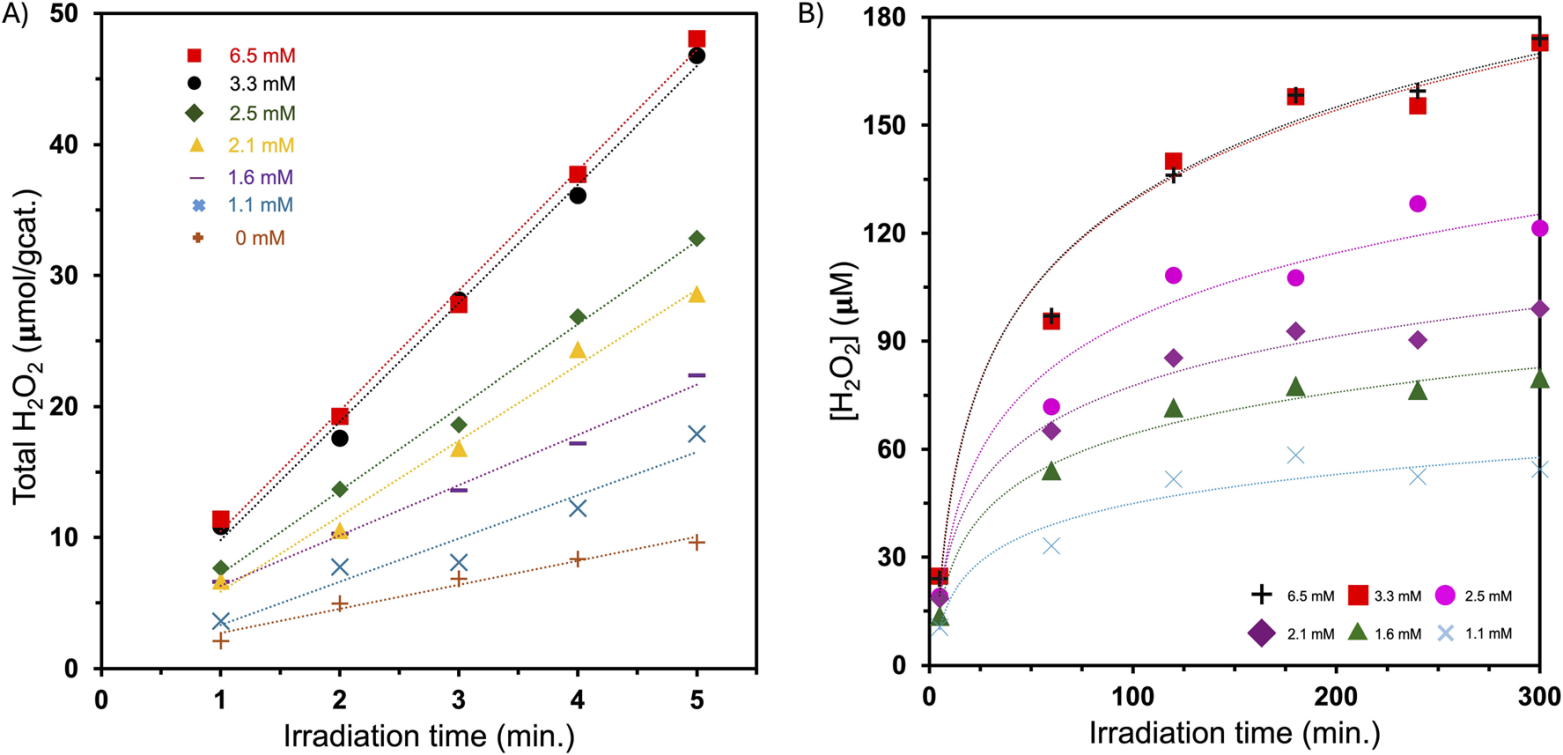
(A) Initial rate of formation of H_2_O_2_ on SiO_2_/NiO–TiO_2_ at varying AgNO_3_ concentrations. (B) [H_2_O_2_] *versus* time at varying AgNO_3_ concentration on SiO_2_/NiO–TiO_2_. Legend: +, 6.5 mM; square, 3.3 mM; circle, 2.5 mM; diamond, 2.1 mM; triangle, 1.6 mM; X, 1.1 mM.

A series of experiments were conducted to assess the initial rate of formation of H_2_O_2_ at varying concentrations of AgNO_3_ (Fig. 4 and Table 1). Aliquots of the reaction solution were extracted at one-minute intervals using a syringe, purified by centrifugation, and reacted with iodide to determine the rate of H_2_O_2_ formation. The SiO_2_/NiO–TiO_2_ catalyst shows an initial rate of formation of H_2_O_2_ as 1.8 μmol g^−1^ min^−1^ and steady state [H_2_O_2_] = 52 μM. Addition of AgNO_3_ to the reaction leads to higher initial rate of formation of H_2_O_2_ up to a limit. In our conditions of 167 mg catalyst (5 mg active phase) in 10 mL of water, the highest initial rate and steady state concentration of hydrogen peroxide were found with a starting concentration of 3.3 mM AgNO_3_. This gave an initial rate of formation of H_2_O_2_ as 9.0 μmol g^−1^ min^−1^ and steady state [H_2_O_2_] = 172 μM. Higher concentrations of AgNO_3_ had very little to no effect on the hydrogen peroxide activity. Below this concentration threshold, we observe a strong dependence of the initial rate and steady state concentration of hydrogen peroxide on AgNO_3_. As an effective electron scavenger, addition of silver nitrate to the system appears to provide a competitive pathway for consuming photogenerated electrons *via* reduction of Ag^+^ to Ag(0). As a result, we observe higher initial rate of formation of H_2_O_2_ and higher steady state concentration of H_2_O_2_ due to the requirement of generating a higher concentration of hydrogen peroxide to effectively compete with silver ion reduction.

**Table 1:** Initial activity and steady state concentration of hydrogen peroxide at varying AgNO_3_ concentration in aqueous solution with SiO_2_/NiO–TiO_2_ catalyst

[AgNO_3_] (mM)	Initial rate, H_2_O_2_ (μmol g^−1^ min^−1^)	Steady state [H_2_O_2_] (μM)
175	8.8	168
6.5	9.1	174
3.3	9.0	172
2.5	6.3	121
2.1	5.7	98
1.6	3.8	80
1.1	3.3	58
0	1.8	52

Photocatalysts materials have been reported to yield hydrogen peroxide from reduction of molecular oxygen or oxidation of water or hydroxide ion.^63,73–75^ Photochemical reactions for hydroxyl radical and hydrogen peroxide were computed for small anatase particles with surface hydroxyls.^76^ The Nosaka group proposed distinct mechanisms for hydroxyl radical formation on anatase and rutile TiO_2_.^77^ On anatase, trapped holes lead to the formation of adsorbed OH radicals, while on rutile, the surface favors O_2_ generation, where H_2_O_2_ is oxidized to O_2_, with hydroxyl radicals produced as a byproduct.^78^ Shi *et al.* investigated O_2_ and H_2_O_2_ formation trends for photoanodes like WO_3_, SnO_2_, TiO_2_, and BiVO_4_ using theoretical and experimental methods.^79^ They found that hydroxyl radical adsorption energy on these surfaces determines the reaction pathway, influencing the O_2_/H_2_O_2_ ratio. Strong hydroxyl radical adsorption favors O_2_ formation, while weak adsorption promotes H_2_O_2_ production. McKay *et al.* reported room temperature water dissociation on the NiO surface, forming adsorbed OH radicals.^80^ We point out that the preponderance of the water splitting literature concerns 4-electron water oxidation to molecular oxygen. On multiple occasions, this difficult reaction has been cited as a bottleneck to solar fuels catalysis.^25,26^ We point out that natural photosynthesis may require a 4-electron water oxidation step to avoid toxic reactive oxygen species that damage the cell. Artificial systems do not have this limitation, and we may re-evaluate the emphasis on designing systems capable of the difficult 4-electron water oxidation step. Alternative pathways that are kinetically less demanding can bypass the difficult bottleneck yet provide a fast pathway to water-splitting. Engineering solutions are anticipated to be vital. If the reactor and catalyst can be configured to carry away hydrogen peroxide from the NiO–TiO_2_ then it should be possible to continuously produce hydrogen peroxide in the system. We expect this to be the work of future studies.

## Conclusion

Multiply decorated NiO–TiO_2_ heteronanocrystals were prepared and the morphology, crystal phase, and heteronanojunctions were established. The NiO–TiO_2_ was supported on high surface area fumed silica and the SiO_2_/NiO–TiO_2_ was subjected to thermal treatment to remove organic ligands. Irradiation of the aqueous suspension of catalyst leads to fast production of hydrogen peroxide; however, as the concentration of hydrogen peroxide increases, it is reduced with the result that the chemical cycle is closed, and steady-state hydrogen peroxide concentration was reached. Together, the results suggest that NiO–TiO_2_ heteronanocrystals are effective materials for exciton generation and separation, that fast catalysis of water oxidation to hydrogen peroxide occurs on the NiO surface of the heteronanocrystal, and fast catalysis for reduction of hydrogen peroxide occurs on TiO_2_. Addition of AgNO_3_ to the reaction leads to higher initial rates of formation and higher steady state concentration of hydrogen peroxide that we propose as due to reduction of Ag^+^ on TiO_2_ competing with reduction of hydrogen peroxide. We predict that engineering inputs such as reactor design and optimization of the reaction conditions could lead to fast, economically relevant total water splitting catalysis.

## Data availability

Data associated with this publication (powder X-ray diffraction and reaction kinetics) may be found on the Open Science Framework at https://www.doi.org/10.17605/OSF.IO/9W2KD.

## Conflicts of interest

The authors declare no competing financial interest.

## Supplementary Material

NA-OLF-D4NA00906A-s001
