# Supplementary material for: Light driven water oxidation on silica supported NiO–TiO2 heteronanocrystals yields hydrogen peroxide
Source: Nanoscale Adv. 2025 Feb 10;7(7):1914–22. doi: 10.1039/d4na00906a (PMC11808566; doi:10.1039/d4na00906a)
Supplement: NA-007-D4NA00906A-s001 [file NA-007-D4NA00906A-s001.pdf]

Supplementary Information (SI) for Nanoscale Advances.
This journal is © The Royal Society of Chemistry 2025

# Light driven water oxidation on silica supported NiO-$TiO_2$ heteronanocrystals yields hydrogen peroxide

Nurul Muttakin,[1] Shelton J.P. Varapragasam,[1] Rashed Mia,[1] Mahfuz A. Swaden,[1] Michael Odlyzko,[2] James D. Hoefelmeyer*[1]

[1] *Department of Chemistry, University of South Dakota, Vermillion, SD 57069*
[2] *Characterization Facility, University of Minnesota, Minneapolis, MN 55455*

* Corresponding author email: james.hoefelmeyer@usd.edu

**Supporting information**

**Contents**


| | |
|---|---|
| **Figure S1.** HRTEM image of NiO-$TiO_2$ heteronanocrystal. | Page 2 |
| **Figure S2. A**) UV-Visible absorption spectra of dilute dispersions of $TiO_2$, $TiO_2$-NiO, and B) Photoluminescence emission spectra ($\lambda_{ex}$ = 300 nm) of anatase $TiO_2$ and $TiO_2$-NiO HNCs. | Page 3 |
| **Figure S3**. Photograph of aqueous suspension of $SiO_2$/NiO-$TiO_2$ (NiO-$TiO_2$ heteronanocrystals supported on fumed silica) after 24 hours illumination with unfiltered light from 150W Xe arc lamp. | Page 4 |
| **Figure S4.** a) Formation of Ag nanoparticles on the aqueous solution after irradiation of light in presence of photocatalyst and b) TEM image of Ag nanoparticles. | Page 5 |
| **Figure S5.** a) Formation of an iron (III) thiocyanate complex ion $Fe(SCN)^{2+}$ from $Fe^{3+}$ and $SCN^-$ in the reaction vessel b) absorbance maxima $\lambda_{max}$= 450 nm of $Fe(SCN)^{2+}$ taken at desired time interval. | Page 6 |
| Analysis of EDS data for NiO-$TiO_2$ heteronanocrystal sample. | Page 7 |

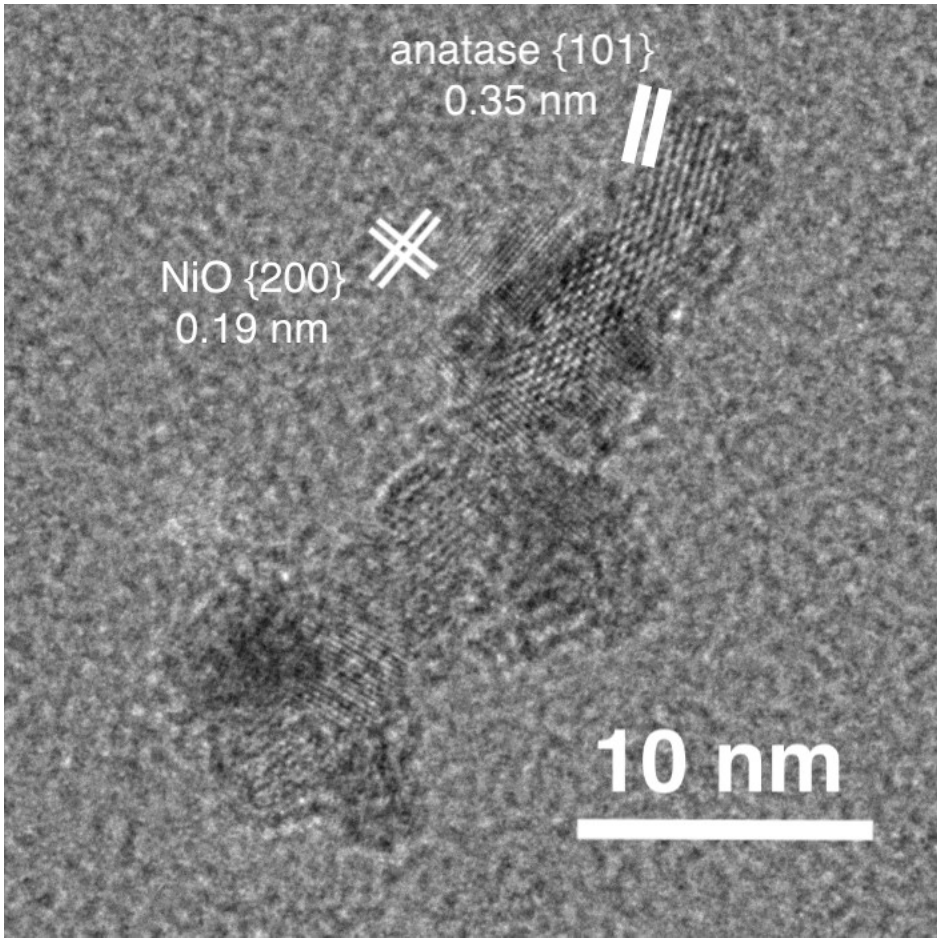


**Figure S1.** HRTEM image of NiO-$TiO_2$ heteronanocrystal.

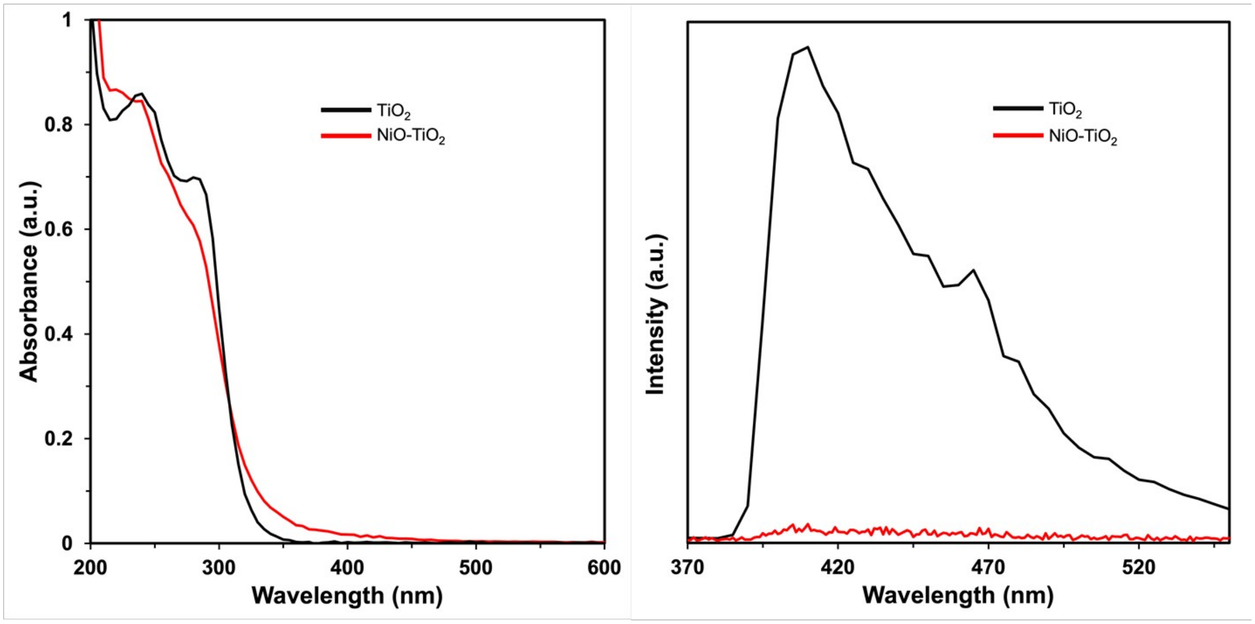


**Figure S2.** A) UV-Visible absorption spectra of dilute dispersions of $TiO_2$, $TiO_2$-NiO, and B) Photoluminescence emission spectra ($\lambda_{ex} = 300$ nm) of *anatase* $TiO_2$ and $TiO_2$-NiO HNCs.

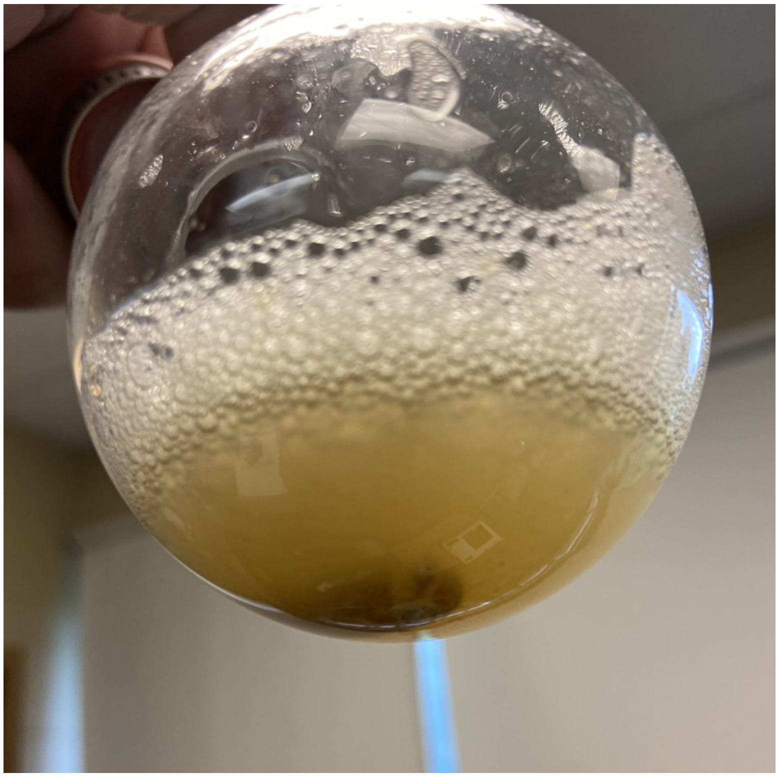

**Figure S3**. Photograph of aqueous suspension of $SiO_2$/NiO-$TiO_2$ (NiO-$TiO_2$ heteronanocrystals supported on fumed silica) after 24 hours illumination with unfiltered light from 150W Xe arc lamp.

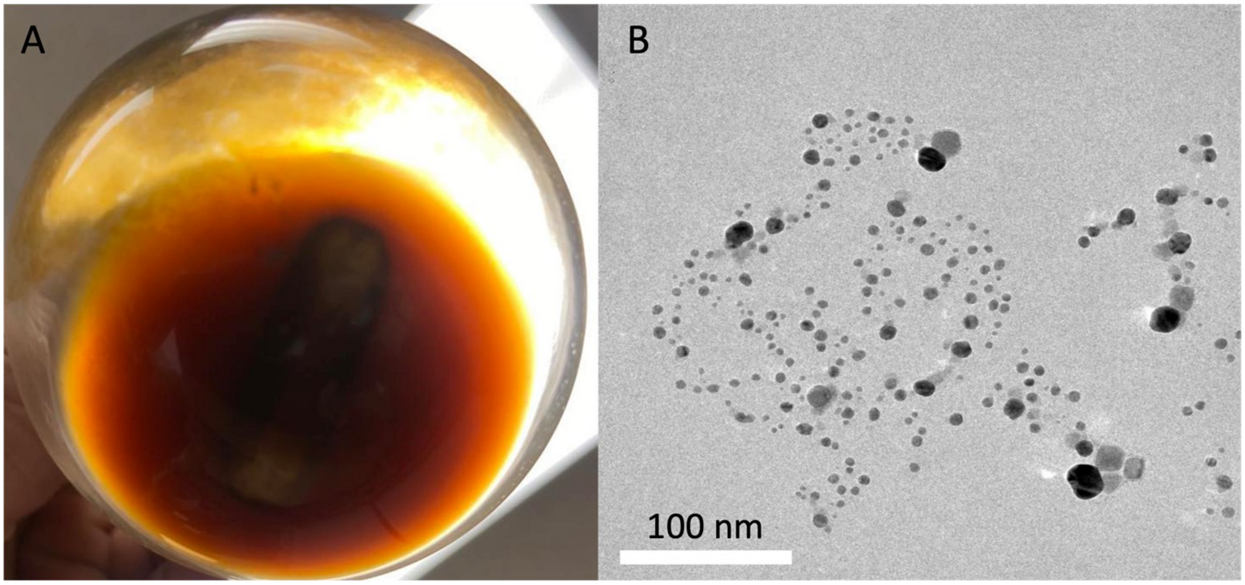


**Figure S4**. Irradiation of $SiO_2$/NiO-$TiO_2$ in dilute aqueous silver nitrate leads to formation of Ag nanoparticles. A) Photograph of the catalyst suspension after irradiation. B) TEM image of Ag nanoparticles.

Alternatively, we demonstrate a fast colorimetric test for hole scavenging upon irradiation of aqueous, anaerobic $Fe^{2+}$ and thiocyanide ion that gives dark red colored solutions upon oxidation to $Fe^{3+}$ (Figure S2). In these conditions, the absorbance at 450 nm abruptly rises, then decreases to a constant value that we presume is due to the back-reaction in which $Fe^{3+}$ is reduced back to $Fe^{2+}$ by photogenerated electrons on $TiO_2$.

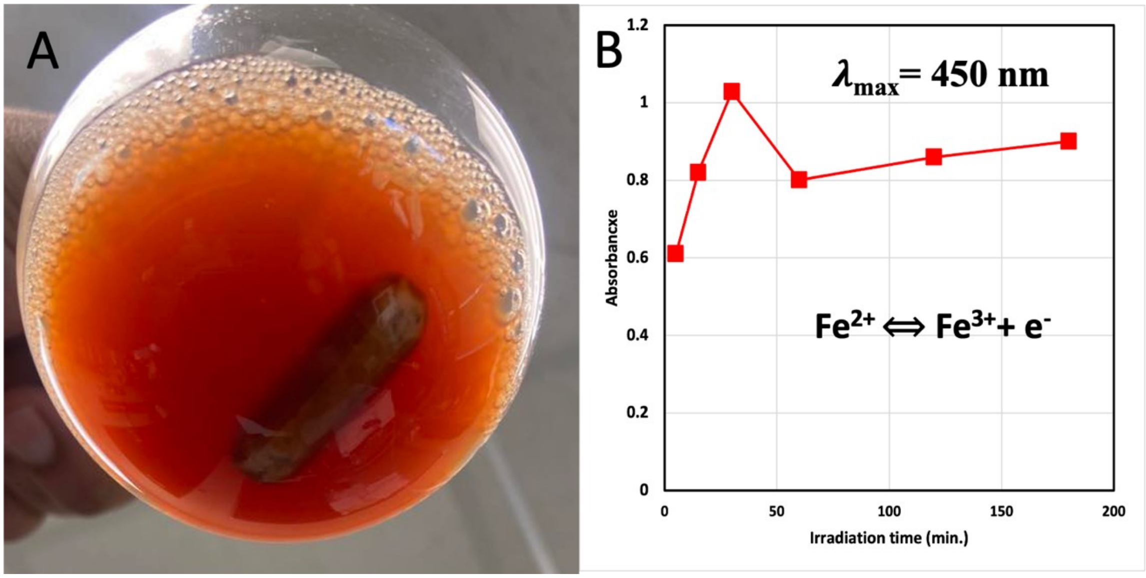


**Figure S5**. a) Formation of an iron (III) thiocyanate complex ion $Fe(SCN)^{2+}$ from $Fe^{3+}$ and $SCN^-$ in the reaction vessel b) absorbance maxima $\lambda_{max}$= 450 nm of $Fe(SCN)^{2+}$ taken at desired time interval.

**Analysis of EDS data for NiO-TiO2 heteronanocrystal sample**.

2023-07-07 11:49:39 Analysis of spectrum: Spectra from Area #1 (Brown-Powell general)

Z Element Family Atomic Fraction (%) Atomic Error (%) Mass Fraction (%) Mass Error (%) Fit error (%)

| Z | Element | Family | Atomic Fraction (%) | Atomic Error (%) | Mass Fraction (%) | Mass Error (%) | Fit error (%) |
|---|---|---|---|---|---|---|---|
| 6 | C | K | 72.91 | 5.19 | 57.99 | 2.48 | 0.09 |
| 7 | N | K | 0.00 | 0.04 | 0.00 | 0.04 | 0.00 |
| 8 | O | K | 18.47 | 3.92 | 19.57 | 4.00 | 0.15 |
| 11 | Na | K | 1.59 | 0.33 | 2.42 | 0.49 | 0.24 |
| 13 | Al | K | 2.02 | 0.42 | 3.60 | 0.73 | 0.23 |
| 14 | Si | K | 0.84 | 0.17 | 1.56 | 0.31 | 0.46 |
| 15 | P | K | 0.21 | 0.04 | 0.43 | 0.08 | 2.18 |
| 19 | K | K | 0.34 | 0.06 | 0.87 | 0.16 | 0.88 |
| 22 | Ti | K | 0.75 | 0.11 | 2.38 | 0.33 | 0.38 |
| 26 | Fe | K | 0.16 | 0.02 | 0.59 | 0.08 | 1.26 |
| 28 | Ni | K | 2.73 | 0.41 | 10.60 | 1.48 | 0.12 |

2023-07-07 11:52:58 Analysis of spectrum: Spectra from Area #1 (Schreiber-Wims oxide-optimized)

Z Element Family Atomic Fraction (%) Atomic Error (%) Mass Fraction (%) Mass Error (%) Fit error (%)

| Z | Element | Family | Atomic Fraction (%) | Atomic Error (%) | Mass Fraction (%) | Mass Error (%) | Fit error (%) |
|---|---|---|---|---|---|---|---|
| 6 | C | K | 69.25 | 5.28 | 52.23 | 2.38 | 0.09 |
| 7 | N | K | 0.00 | 0.04 | 0.00 | 0.04 | 0.00 |
| 8 | O | K | 19.87 | 4.25 | 19.97 | 4.10 | 0.15 |
| 11 | Na | K | 1.83 | 0.39 | 2.64 | 0.54 | 0.24 |
| 13 | Al | K | 2.35 | 0.50 | 3.99 | 0.81 | 0.23 |
| 14 | Si | K | 0.99 | 0.20 | 1.74 | 0.34 | 0.46 |

| | | | | | | | |
|---|---|---|---|---|---|---|---|
| 15 | P | K | 0.25 | 0.05 | 0.48 | 0.09 | 2.18 |
| 19 | K | K | 0.41 | 0.08 | 1.00 | 0.18 | 0.88 |
| 22 | Ti | K | 0.94 | 0.14 | 2.82 | 0.40 | 0.38 |
| 26 | Fe | K | 0.21 | 0.03 | 0.75 | 0.11 | 1.26 |
| 28 | Ni | K | 3.90 | 0.60 | 14.39 | 2.03 | 0.12 |
